# Correction: BMC Emerg Med, Vol 21

**DOI:** 10.1186/s12873-023-00822-w

**Published:** 2023-05-23

**Authors:** Zhenyu Luo

**Affiliations:** Guangyuan Central Hospital, Guangyuan, Sichuan, China

**Correction**

The original articles [1, 2] mistakenly misspell Guangyuan Central Hospital as ‘Guanyuan’ in Affiliation #1. The authors wish to note the correct spelling in this Correction article.
